# Clinical characteristics and risk score for poor clinical outcome of acute ischemic stroke patients treated with intravenous thrombolysis therapy

**DOI:** 10.1002/brb3.1251

**Published:** 2019-03-11

**Authors:** Yun‐hua Yue, Zhi‐zhang Li, Liang Hu, Xiao‐qiong Zhu, Xu‐shen Xu, Hong‐xian Sun, Zhi‐wen Wan, Jie Xue, De‐hua Yu

**Affiliations:** ^1^ Department of Neurology Yangpu Hospital Tongji University School of Medicine Shanghai China; ^2^ Department of General Medicine Yangpu Hospital Tongji University School of Medicine Shanghai, Shanghai China

**Keywords:** outcome, stroke, tissue plasminogen activator

## Abstract

**Background:**

Tissue plasminogen activator (t‐PA) is an effective therapy for acute ischemic stroke, but some patients still have poor clinical outcome. In this study, we investigated clinical characteristics of stroke patients and determined predictors for poor clinical outcome in response to t‐PA treatment.

**Methods:**

Clinical data from 247 patients were retrospectively reviewed. Clinical parameters that were associated with survival of patients were analyzed. Areas under receiver operating characteristic curves (ROC) were used to determine the feasibility of using various combinations of the clinical parameters to predict poor clinical response. The clinical outcome was defined according to the changes in Modified Rankin Scale.

**Results:**

Overall, 145 patients had improved/complete recovery, 73 had no change, and 29 had worsening conditions or died during the in‐clinic period. A univariate analysis showed that baseline characteristics including age, CRP, blood glucose level, systolic blood pressure, and admission NIHSS were significantly different (*p < *0.05) among patients with different clinical outcome. A further multivariate analysis was then performed. Variables associated with poor clinical outcome (worsening/death) (*p* < 0.1) were included in the logistic regression model. Four parameters were retained in the model: Age, CRP, Blood glucose level, and Systolic blood pressure (ACBS). To allow a convenient usage of the ACBS classifier, the parameters were put into a scoring system, and the score at 7.7 was chosen as a cut‐off. The ROC curve of this ACBS classifier has an area under the curve (AUC) of 0.7788, higher than other individual parameters. The ACBS classifier provided enhanced sensitivity of 69.2% and specificity of 74.3%.

**Conclusion:**

The ACBS classifier provided a satisfactory power in estimating the patients’ clinical outcome. After further validating, the classifier may provide important information to clinicians for making clinical decisions.

## 1 INTRODUCTION

Stroke is the leading cause of disability among adults. It is caused by thrombotic or embolic occlusion of a cerebral artery. Each year, about 800,000 strokes occurred; and of all strokes, 87% are ischemic in origin (Roger et al., 2011). The risk of stroke is higher among elderly than youngsters (Rosamond et al., 1999). Many of the stroke survivors continue to experience functional deficits that diminished their quality of life. In Framingham study, almost half of all elderly stroke survivors had moderate to severe neurological deficits (Kelly‐Hayes et al., 2003). Because of an increase in life expectancy, the number of individuals at risk for ischemic stroke is expected to increase, making the management of stroke disability among elderly an important public health concern.

Tissue plasminogen activator (t‐PA) is a US Food and Drug Administration‐approved drug for treatment of ischemic stroke. It is a serine protease that enhances the conversion of inactive plasminogen to active plasmin. Plasmin acts on fibrin clots, resulting in dissolution and lysis. Studies suggested that the administration of t‐PA within 3 hr after the onset of stroke increases the probability of favorable clinical outcome (Wechsler, 2011). However, only a small fraction of potentially eligible stroke patients is receiving t‐PA therapy. In US, it is estimated that the rate of t‐PA use averages not more than 2% (Katzan et al., 2000). In China, the rate of t‐PA usage was only about 1.6% (Dong et al., 2017). The difficulty in predicting the clinical outcome of patients receiving t‐PA treatment is hampering the widespread usage of t‐PA by clinicians.

A prognostic model that would allow an early estimation of clinical outcomes of patients receiving t‐PA treatment is in need. A handy prognostic model should contain variables that are readily available in clinical settings for all patients. To date, there have been limited prognostic models for stroke recovery, and the predictive power was not satisfactory (Counsell & Dennis, 2001). There were other models that relied on imaging variables, which may not be available for all patients (Baird et al., 2001; Johnston, Connors, Wagner, & Haley, 2003; Johnston et al., 2000). In the current study, we investigated different baseline clinical characteristics that may associate with the poor clinical outcome of stroke patients as assessed by Modified Rankin Scale (MRS). These patients likely would not benefit from the t‐PA therapy.

## 2 PATIENTS AND METHODS

### 2.1 Patient characteristics

A total of 247 patients with acute ischemic stroke were included in this study, and their clinical data were retrospectively reviewed. These patients were admitted to the Department of Neurology, Yangpu Hospital, Tongji University School of Medicine between January 2016 and December 2017. On arrival in the emergency room, patients underwent standard neurological and cardiological examinations. Blood chemistry, vital signs, and CT scans of the brain were obtained before the start of treatment. The CT scans were reviewed by a neuroradiologist with extensive experience in acute stroke. Previous and concomitant diseases were recorded.

All of the patients were treated with t‐PA at a dose of 0.9 mg/kg. National Institute of Health Stroke Scale (NIHSS) score was assessed at various time points during the clinical stay. Patients with admission NIHSS ≥20 were considered as severe, 4–19 were moderate, and <4 represented mild or normal. MRS was assessed at admission and at discharge. We defined the clinical outcome according to the differences in admission MRS and discharge MRS. Patients with a lower MRS (or MRS = 0) at discharge were defined as “improved/complete cure,” those with the same MRS were defined as “no change,” and those with a higher MRS (or died after treatment) were defined as “worsening/death.” Adverse events of hemorrhage, including symptomatic intracerebral hemorrhage, were identified. The study was approved by the Hospital's ethics committee.

### 2.2 Statistical analyses

All statistical analyses were carried out by SAS9.3. Continuous variables were expressed as mean ± SD, whereas categorical variables were expressed as numbers (percentages). A value of *p* < 0.05 was considered statistically significant. For comparisons of three or more groups with data normally distributed, one‐way analysis of variance was used; otherwise, Kruskal–Wallis test was used.

A univariate analysis was performed to compare variables among the groups: Improved/complete cure, No change, and Worsening/death. Variables associated with poor clinical outcome (worsening/death) (*p* < 0.1) were included in the logistic regression model. In the meantime, variable selection was performed to balance the predictive power and complexity of the model. The receiver operating characteristic (ROC) curve and the area under the curve (AUC) were used to determine the feasibility of using clinical parameters as a classifier to predict treatment response of patients. Mann–Whitney test was used to compare the differences of AUC between various parameters. The Youden's Index was used to identify the optimal cut‐off point. Sensitivity, specificity, and confidence interval calculations were performed using standard procedures.

## 3 RESULTS

We evaluated a total of 247 patients (145 males, 102 female) with acute ischemic stroke; all of them were treated with intravenous t‐PA. Demographic data, baseline clinical findings, and medical history were shown in Table 1. The mean age of the cohort was 69.78 ± 13.62 years. Patients arrived at the emergency department after stroke onset in 1.64 ± 2.21 hr. The time elapsed between symptom onset and t‐PA treatment was 2.72 ± 2.56 hr. Baseline clinical assessment revealed that 75.3% of the patients had hypertension. The median NIHSS score of the cohort at admission was 3.

**Table 1:** Baseline characteristics of patients (*n* = 247) with acute ischemic stroke

Characteristics	Values	Baseline characteristics	Values
Age (year), mean ± SD	69.78 ± 13.62	Medical history	
Male, *n* (%)	145 (58.70)	Hypertension, *n* (%)	186 (75.30)
CRP (mg/L), median (IQR)	0 (0, 6)	Diabetes, *n* (%)	71 (28.74)
Creatinine (μmol/L), median (IQR)	73 (60,92)	Atrial fibrillation, *n* (%)	41 (16.60)
Uric acid (μmol/L), median (IQR)	293 (211, 371)	Renal insufficiency, *n* (%)	11 (4.45)
Blood glucose (mmol/L), median (IQR)	6.76 (5.74, 8.68)	Coronary heart Disease, *n* (%)	20 (8.10)
Homocysteine (μmol/L), median (IQR)	16.05 (11.79, 21.00)	Lung infection, *n* (%)	1 (0.40)
Systolic blood pressure (mmHg), median (IQR)	154 (142, 170)	Cancer, *n* (%)	2 (0.81)
Diastolic blood pressure (mmHg), median (IQR)	86 (76, 95)	Cerebral infraction, *n* (%)	12 (4.86)
NIHSS, median (IQR)	3 (2, 7)	Other medical history, *n* (%)	35 (14.17)

NIHSS data were completed at baseline in 247 (100%), at 2 hr in 246 (99.6%), at 24 hr in 232 (93.9%), at 2 days in 221 (89.5%), at 3 days in 211 (85.4%), at 7 days in 196 (79.4%), and at 14 days in 81 (32.8%) patients. MRS data were completed at baseline and at discharge. Higher number of patients had a mild severity (score 0–1) of MRS at discharge (*n* = 179) than at the baseline (*n* = 124). Overall, 145 patients had improved/complete recovery, 73 had no changes, and 29 had the outcome of worsening/died.

It has been known that the major complication of thrombolytic therapy for acute stroke is hemorrhage. In the current cohort, the reported treatment‐related hemorrhage included oral (*n* = 18), gastrointestinal (*n* = 2), and intracranial hemorrhage (*n* = 8). Most of the related adverse events (AEs) were mild (*n* = 14) or moderate (*n* = 12) in intensity. A total of eight related AEs were severe, including three events of deaths (these three patients had symptomatic hemorrhage).

A univariate analysis was performed for the available variables, and the results showed that baseline characteristics including age, CRP, blood glucose level, systolic blood pressure, and admission NIHSS were significantly different (*p < *0.05) among patients with different clinical outcome (Table 2). A further multivariate analysis was then performed. Variables associated with poor clinical outcome (worsening/death) (*p* < 0.1) were included in the logistic regression model. In the meantime, variable selection was performed. After balancing the predictive power and complexity of the model, four parameters were retained: Age, CRP, Blood glucose level, and Systolic blood pressure (ACBS). These parameters were statistically significant among groups, and their predictive power was high when used in combination. Also, these were objective parameters (when compared to NIHSS) and could be obtained easily in clinical setting. Recognizing the relatively wide 95% CI of the data, several models were established (Table 3).

**Table 2:** Univariate analysis of baseline characteristics and clinical outcome

Baseline characteristics	Improved/complete cure, *n* = 145	No change, *n* = 73	Worsening/death	*p*
Age (year), mean ± SD	69.17 ± 13.24	68.05 ± 14.57	77.14 ± 10.79	0.001
80 ≤ age	38 (26.39)	21 (28.77)	15 (51.72)	0.029
70 ≤ age < 80	27 (18.75)	13 (17.81)	5 (17.24)	
60 ≤ age < 70	46 (31.94)	16 (21.92)	8 (27.59)	
Age <60	33 (22.92)	23 (31.51)	1 (3.45)	
Gender, *n* (%)				
Male	86 (59.31)	40 (54.79)	19 (65.52)	0.595
Female	59 (40.69)	33 (45.21)	10 (34.48)	
Hypertension, *n* (%)	105 (72.41)	59 (80.82)	22 (75.86)	0.396
Diabetes, *n* (%)	41 (28.28)	19 (26.03)	11 (37.93)	0.479
Atrial fibrillation, *n* (%)	22 (15.17)	16 (21.92)	3 (10.34)	0.283
Renal insufficiency, *n* (%)	5 (3.45)	2 (2.74)	4 (13.79)	0.072
Coronary heart disease, *n* (%)	13 (8.97)	5 (6.85)	2 (6.90)	0.837
Lung infection, *n* (%)	0 (0.00)	1 (1.37)	0 (0.00)	0.413
Cancer, *n* (%)	0 (0.00)	1 (1.37)	1 (3.45)	0.083
Cerebral Infraction, *n* (%)	6 (4.14)	4 (5.48)	2 (6.90)	0.753
Other medical history, *n* (%)	17 (11.72)	10 (13.70)	8 (27.59)	0.081
Admission CRP, median, (IQR)	0 (0,6)	0 (0,6)	5 (0,20.89)	0.005
16.5 ≤ CRP	12 (8.45)	6 (8.45)	9 (31.03)	0.030
7 ≤ CRP < 16.5	15 (10.56)	8 (11.27)	2 (6.90)	
0 ≤ CRP < 7	115 (80.99)	57 (80.28)	18 (62.07)	
Creatinine (μmol/L), median (IQR)	74 (62,95)	72 (54,84)	72.5 (58.5,89)	0.206
Uric acid (μmol/L), median (IQR)	290 (205,371)	309 (236,381)	305 (54.84,369)	0.655
Blood Glucose (mmol/L), median (IQR)	6.47 (5.64,8.63)	6.77 (5.65,8.23)	8.36 (6.62,10.93)	0.008
9 ≤ glucose	30 (21.58)	13 (18.31)	12 (44.44)	0.054
7.5 ≤ glucose < 9	15 (10.79)	9 (12.68)	4 (14.81)	
0 < glucose < 7.5	94 (67.63)	49 (69.01)	11 (40.74)	
Homocysteine (μmol/L), median (IQR)	16.46 (11.64,21)	14.6 (10.82,18.58)	20 (14.43,26.98)	0.058
Systolic blood pressure (mmHg), median (IQR)	151 (140,166)	154.5 (143,170)	166 (148,177.5)	0.049
165 ≤ systolic blood pressure	37 (25.87)	22 (31.43)	16 (57.14)	0.024
155 ≤ systolic blood pressure < 165	25 (17.48)	13 (18.57)	4 (14.29)	
0 < systolic blood pressure < 155	81 (56.64)	35 (50.00)	8 (28.57)	
Diastolic blood pressure (mmHg), median (IQR)	85 (76,93)	88 (77,98)	85.5 (75.5,95.5)	0.337
Admission NIHSS, median (IQR)	3 (2,7)	3 (2,5)	4 (3,9)	0.032
20 ≤ NIHSS	8 (5.52)	4 (5.48)	4 (13.79)	0.082
4 ≤ NIHSS < 20	58 (40.00)	26 (35.62)	16 (55.17)	
0 ≤ NIHSS < 4	79 (54.48)	43 (58.90)	9 (31.03)	

**Table 3:** Multivariate analysis of occurrence of worsening/death after treatment in patients with different clinical outcome

Model	Independent variables	Levels (risk factors)	Odds ratio	95% CI		*p*
				Lower limit	Upper limit	
Model 1	Gender	Male versus female	1.141	0.400	3.254	0.805
	Renal insufficiency	Absence versus presence	0.220	0.035	1.398	0.109
	Cancer	Absence versus presence	0.507	0.013	19.172	0.714
	Age	80 ≤ age versus age <60	5.772	0.650	51.238	0.273
		70 ≤ age < 80 versus age <60	5.014	0.474	53.045	0.532
		60 ≤ age < 70 versus age <60	5.662	0.630	50.909	0.316
	CRP	16.5 ≤ CRP versus 0 ≤ CRP < 7	5.694	1.649	19.665	0.008
		7 ≤ CRP < 16.5 versus 0 ≤ CRP < 7	0.868	0.166	4.548	0.227
	Blood glucose	9 ≤ blood glucose versus 0 < blood glucose < 7.5	2.309	0.780	6.835	0.521
		7.5 ≤ blood glucose < 9 versus 0 < blood glucose < 7.5	2.631	0.660	10.498	0.411
	SBP	165 ≤ SBP versus 0 < SBP < 155	5.906	1.869	18.667	0.006
		155 ≤ SBP < 165 versus 0 < SBP <155	1.662	0.393	7.025	0.570
	NIHSS	20 ≤ NIHSS versus 0 ≤ NIHSS < 4	1.867	0.334	10.445	0.678
		4 ≤ NIHSS < 20 versus 0 ≤ NIHSS < 4	1.770	0.601	5.211	0.644
Model 2	Age	Unit = 1	1.044	1.007	1.082	0.019
	CRP	Unit = 1	1.013	0.999	1.027	0.068
	Blood glucose	Unit = 1	1.091	0.967	1.231	0.158
	SBP	Unit = 1	1.024	1.004	1.044	0.016
Model 3	Age	80 ≤ age versus age <60	6.039	0.719	50.746	0.253
		70 ≤ age < 80 versus age <60	6.207	0.637	60.431	0.326
		60 ≤ age < 70 versus age <60	5.536	0.628	48.843	0.394
	CRP	16.5 ≤ CRP versus 0 ≤ CRP < 7	6.732	2.108	21.503	0.002
		7 ≤ CRP < 16.5 versus 0 ≤ CRP < 7	0.912	0.179	4.638	0.200
	Blood glucose	9 ≤ blood glucose versus 0 < blood glucose < 7.5	2.512	0.914	6.902	0.352
		7.5 ≤ blood glucose < 9 versus 0 < blood glucose < 7.5	2.387	0.620	9.192	0.531
	SBP	165 ≤ SBP versus 0 < SBP < 155	5.654	1.888	16.931	0.006
		155 ≤ SBP < 165 versus 0 < SBP < 155	1.783	0.428	7.422	0.664

Note

Model 1, the characteristic variables of *p* < 0.1 in the univariate analysis were all used as independent variables, and the logistic regression model was established by using clinical outcome of worsening/death after treatment. Model 2: Age, CRP, blood glucose, SBP were selected as independent variables. Model 3: Age, CRP, blood glucose, and SBP were selected as an independent variable.

The four variables, age, CRP, blood glucose, and SBP, were continuous variables and might not be convenient to use in a regression model. The parameters were thus put into a scoring system (Table 4). The levels were first assigned on the basis of the cut‐off of the single parameter, and then adjusted in combination with other parameters until the predictive power of the classifier became satisfactory (higher predictive value than the single parameter). The score at 7.7 was chosen as a cut‐off, as it has the highest Youden's Index. Risk of the poor clinical outcome increased with higher scores.

**Table 4:** ACBS scoring system

Characteristics	Criteria	Score
Age (year)	<60	0
	60–69	1
	70–79	2
	≥80	3
CRP (mg/L)	<7	1
	7–16.4	2
	≥ 16.5	3
Blood glucose level (mmol/L)	<7.5	1
	7.5–8.9	2
	≥9	3
Systolic blood pressure (mmHg)	<155	1
	155–164	2
	≥165	3

**Figure 1:**
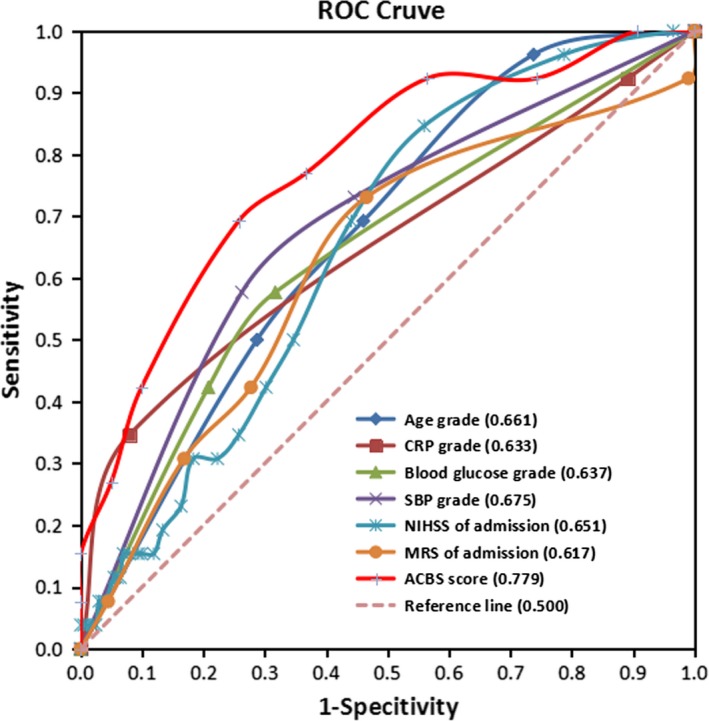
Receiver operating characteristic curves for age, CRP, blood glucose grade, SBP grade, baseline NIHSS, baseline MRS, and ACBS classifier with respect to clinical outcome

The ROC curve of this ACBS classifier has an AUC of 0.7788, while other individual parameters had smaller AUC, including age (AUC = 0.6617), CRP (AUC = 0.6337), blood glucose grade (AUC = 0.6371), SBP grade (AUC = 0.6753), admission NIHH (AUC = 0.6510), and admission MRS (AUC = 0.6173) (Table 5 and Figure 1). The ACBS classifier provided enhanced sensitivity of 69.2% and specificity of 74.3%.

**Table 5:** Comparisons of AUC between ACBS classifier and various parameters

Indicators	AUC	95% CI		Difference*	*p* *
		Lower limit	Upper limit		
ACBS score	0.7788	0.6831	0.8744	–	–
Age	0.6617	0.5667	0.7567	−0.1451	0.0020
CRP	0.6337	0.5267	0.7406	−0.1417	0.0182
Blood glucose	0.6371	0.5306	0.7435	−0.1035	0.0109
SBP	0.6753	0.5702	0.7803	−0.1278	0.0453
NIHSS of admission	0.6510	0.5541	0.7479	−0.1615	0.0358
MRS of admission	0.6173	0.4991	0.7355	−0.2788	0.0153
Reference line	0.5000	0.5000	0.5000	−0.1451	<0.0001

* The differences in AUC and *p*‐value compared to ACBS.

## 4 DISCUSSION

The current study showed that the combination of age, CRP, baseline SBP, and blood glucose was a classifier that could predict patients with high risk of poor response to t‐PA treatment. Comparing to other predictive models (Baird et al., 2001; Johnston et al., 2003, 2000), the ACBS scoring system is simple to use. It is based on commonly assessed clinical parameters for stroke patients.

Several analyses from observational studies as well as randomized clinical trials reported the predictive value of age (Fiorelli et al., 1995; Generalized efficacy of t‐PA for acute stroke, 1997; Johnston et al., 2003). In the current study, patients with worsening/death outcome after treatment were significantly older. Age was also a variable in the ACBS classifier predicting the clinical outcome. The DRAGON score, which includes age in the scoring system, predicts the functional outcome of patients as assessed by MRS as well (Kent, 2012). Indeed, age was also a variable in different models that predict the risk of hemorrhage after t‐PA (Lou et al., 2008; Lyden, 2012; Menon et al., 2012). Overall, the data suggested that age is an important characteristic that has to be considered for clinical decision.

Our findings indicated that high blood glucose level was associated with poor clinical outcome of patients. Several studies also showed that hyperglycemia was related to poor outcome of acute ischemic stroke patients (Fuentes, 2010; Stead et al., 2009; Williams et al., 2002). The relationship between high blood glucose level and severity of ischemic stroke can be explained by the increase of lactate production in the ischemic region. The production lead to generation of hydrogen ions and disruption of intracellular pH homeostasis. As a result, some reactions and enzyme systems that are essential to cellular viability were interrupted (Lindsberg & Roine, 2004; Pulsinelli, Waldman, Rawlinson, & Plum, 1982; Rehncrona, Rosen, & Siesjo, 1981). In the current cohort, about 35% of patients had high blood glucose level (>7.5 mmol/L). Initiation of intensive insulin therapy has been suggested for stroke patients, but currently available data fail to identify the clinical benefits of the therapy, for example, the UK Glucose Insulin in Stroke Trial (GIST‐UK) showed no significant clinical benefit associated with insulin therapy in 933 patients with stroke (Gray et al., 2007).

High blood pressure is common in acute stroke patients. Several studies suggested a U‐shaped relationship between baseline blood pressure and poor outcome of ischemic stroke (Leonardi‐Bee, Bath, Phillips, & Sandercock, 2002; Vemmos et al., 2004). Both high blood pressure and low blood pressure were prognostic for poor outcome. The analyses of data from the thrombolysis implementation and monitor of AIS in China (TIMS‐China) showed that a higher first 2 hr systolic blood pressure was related to symptomatic intracerebral hemorrhage. A proper control of systolic blood pressure for the first 2 hr was thus recommended to decrease the risk of hemorrhage (Wu, 2016). Our study also supported the notion that high systolic blood pressure at admission was related to poor clinical outcome among ischemic stroke subjects.

Several studies have reported the predictive value of plasma CRP concentrations. Elevated CRP concentration predicted poor survival and poor functional outcome of patients with acute ischemic stroke (Mazaheri, Reisi, Poorolajal, & Ghiasian, 2018; Muir, Weir, Alwan, Squire, & Lees, 1999). It was suggested that CRP concentration may correlate with the degree of inflammation directly consequent to cerebral infraction. There were some studies, however, reported that baseline CRP failed to predict clinical outcomes (Karlinski et al., 2014; Topakian, Strasak, Nussbaumer, Haring, & Aichner, 2008). Our results supported the usefulness of baseline CRP as a predictive marker of poor clinical outcome.

Our study has some limitations. The study populations of the current study represent hospital‐based cohorts; unselected patients in different clinical settings are needed for validating the model. Also, the number of patients is limited for establishing a prognostic model. This is a retrospective study, therefore our findings await replication in a prospective cohort to determine the best approach to manage patients with high score in the ACBS scoring system. The cut‐off criteria for the clinical variables will need to be further verified. In summary, we evaluated the baseline clinical characteristics of stroke patients associated with patient outcome as assessed by MRS. The ACBS classifier provided a satisfactory sensitivity and specificity in estimating the patients’ response to t‐PA treatment. After further validating, the classifier may provide important information to clinicians when discussing treatment options with patients and their families.

## 5 CONCLUSION

The ACBS classifier provided a satisfactory power in estimating the patients’ clinical outcome. After further validating, the classifier may provide important information to clinicians for making clinical decisions.

## INFORMED CONSENT

All the patients gave their written information consent.

## ETHICAL APPROVAL

The study was approved by the Hospital's ethics committee.

## CONFLICT OF INTEREST

All the authors declare that they have no conflict of interest.
